# Silver Nanoparticle-Embedded Hydrogels for Electrochemical Sensing of Sulfamethoxazole Residues in Meat

**DOI:** 10.3390/molecules29061256

**Published:** 2024-03-12

**Authors:** Yuanxi Deng, Ningning Yang

**Affiliations:** College of Food and Bioengineering, Bengbu University, Bengbu 233030, China; ynn@bbc.edu.cn

**Keywords:** cellulose, silver nanoparticles, sulfamethoxazole, voltammetry, antibiotic residues, meat, food safety, nanocomposite sensor

## Abstract

A disposable electrochemical sensor based on silver nanoparticle-embedded cellulose hydrogel composites was developed for sensitive detection of sulfamethoxazole residues in meat samples. Scanning electron microscopy confirmed the porous structure of the cellulose matrix anchored with 20–50 nm silver nanoparticles (AgNPs). Fourier transform infrared spectroscopy and X-ray diffraction verified that the metallic AgNPs coordinated with the amorphous cellulose chains. At an optimum 0.5% loading, the nanocomposite sensor showed a peak-to-peak separation of 150 mV, diffusion-controlled charge transfer kinetics, and an electron transfer coefficient of 0.6 using a ferro/ferricyanide redox probe. Square-wave voltammetry was applied for sensing sulfamethoxazole based on its two-electron oxidation peak at 0.72 V vs. Ag/AgCl in Britton–Robinson buffer of pH 7.0. A linear detection range of 0.1–100 μM sulfamethoxazole was obtained with a sensitivity of 0.752 μA/μM and limit of detection of 0.04 μM. Successful recovery between 86 and 92% and less than 6% RSD was achieved from spiked meat samples. The key benefits of the proposed disposable sensor include facile fabrication, an antifouling surface, and a reliable quantification ability, meeting regulatory limits. This research demonstrates the potential of novel cellulose–silver nanocomposite materials towards developing rapid, low-cost electroanalytical devices for decentralized on-site screening of veterinary drug residues to ensure food safety.

## 1. Introduction

The occurrence of veterinary drug residues such as antibiotics and growth promoters in meat has become a major public health concern [1,2,3,4]. Sulfonamides (SMX) are one of the most commonly used veterinary antibiotics for food-producing animals to treat bacterial infections and facilitate growth promotion [5,6,7]. However, their rampant use has led to the emergence of antibiotic-resistant bacteria and also leaves harmful residues in animal food products intended for human consumption [8].

SMX is a frequently detected antibiotic residue belonging to the sulfonamides class. Studies across the world have reported the presence of SMX residues exceeding permissible limits in meat samples [9,10]. Prolonged human exposure to SMX residues through meat consumption can lead to allergic reactions, disruption of the intestinal flora balance, promotion of antimicrobial resistance, and potential carcinogenic effects [11,12]. Therefore, regulatory authorities have set maximum residual limits of 100 μg/kg for SMX in meat [13]. However, the overuse of SMX in livestock rearing calls for the development of rapid analytical techniques to stringently monitor SMX residues in meat products [14].

Conventional methods for detecting antibiotic residues include microbiological and immunoassay techniques, as well as chromatographic techniques coupled to mass spectrometry [15,16]. However, these laboratory-based methods require extensive sample pretreatment, sophisticated instruments, and trained personnel. The demand for decentralized on-site testing has motivated research focused on developing cost-effective, field-portable analytical devices. In this context, electrochemical biosensors have shown tremendous potential for rapid, sensitive detection of food contaminants [17,18,19,20,21,22,23]. Furthermore, modern microfabrication approaches have enabled the transition from bulky electrodes to miniaturized disposable electrochemical sensors.

Recent advances in nanotechnology have stimulated interest in utilizing nanomaterials for enhancing the performance of electrochemical sensors. The excellent conductivity, high surface area and catalytic properties of nanomaterials can improve electron transfer kinetics and sensitivity [24]. Cellulose is an abundant biopolymer that has been widely researched for developing functional nanocomposites using metal nanoparticles as nanofillers [25,26]. This study aims to develop a disposable electrochemical sensor using silver nanoparticle-decorated cellulose hydrogel composites. The gel matrix provides a biocompatible microenvironment and stabilizes the nanoparticles within its porous network. Additionally, the hydrogel composite facilitates the diffusion of analyte molecules to the sensor interface for rapid electron exchange reactions. The sensor is applied for sensitive detection of SMX residues in meat samples using voltammetric analysis. The unique composition and surface architecture of the nanocomposite sensor holds promise for enabling rapid, on-site screening of antibiotic contamination to ensure food safety.

## 2. Results and Discussion

**Figure 1:**
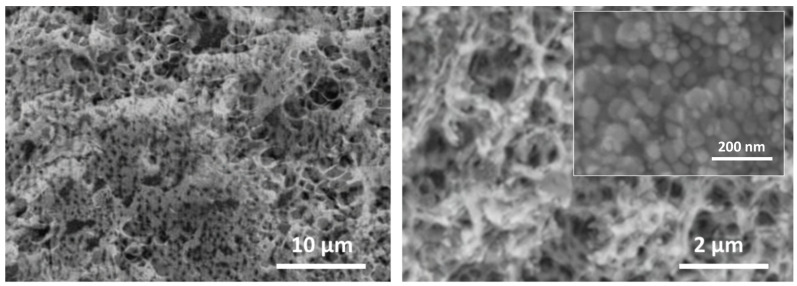
SEM micrographs showing porous morphology of the CS-AgNPs composite hydrogel under different magnifications.

A scanning electron microscope (SEM, JSM-6490LA, JEOL, Tokyo, Japan) was utilized to examine the microstructure of the CS-AgNPs composite hydrogel. Figure 1 shows the SEM images captured at different magnifications, illustrating a highly porous network structure. The cellulose matrix contains micropores in the size range of 1–5 μm, resulting from extensive hydrogen bonding interactions between the polymeric chains during gelation [27]. The pore walls are decorated with AgNPs of 20–50 nm diameter, which are anchored to the electron-dense regions.

**Figure 2:**
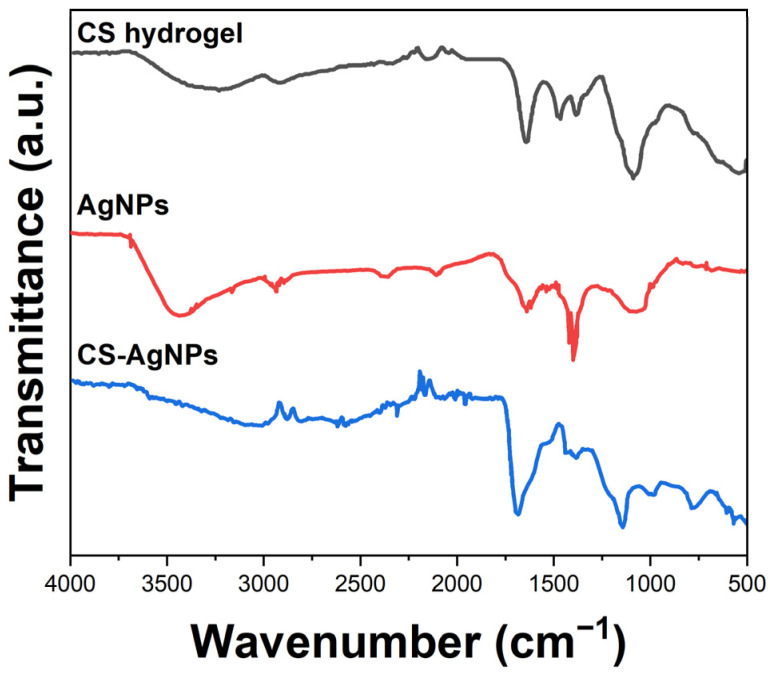
FTIR spectra of CS hydrogel, AgNPs, and CS-AgNPs.

The interaction between cellulose and the AgNPs was investigated by Fourier transform infrared spectroscopy (FTIR, PerkinElmer, Waltham, MA, USA). The FTIR spectrum of cellulose hydrogel (Figure 2) displays a broad peak at 3200–3500 cm^−1^, corresponding to the hydroxyl groups. The bands at 2850–3000 cm^−1^ are attributed to C-H stretching vibrations [28]. The intense peak at 1000–1100 cm^−1^ relates to C-O-C glycosidic ether bonds. In the spectrum of AgNPs, signals at 1550, 1380, and 780 cm^−1^ corroborate the presence of AgNPs [29]. The composite hydrogel spectrum shows additional bands at 1560 and 1390 cm^−1^, associated with carbonyl and carboxyl groups [30]. The shift in peak positions indicates coordination bonding between oxygen functionalities on cellulose and the AgNPs.

**Figure 3:**
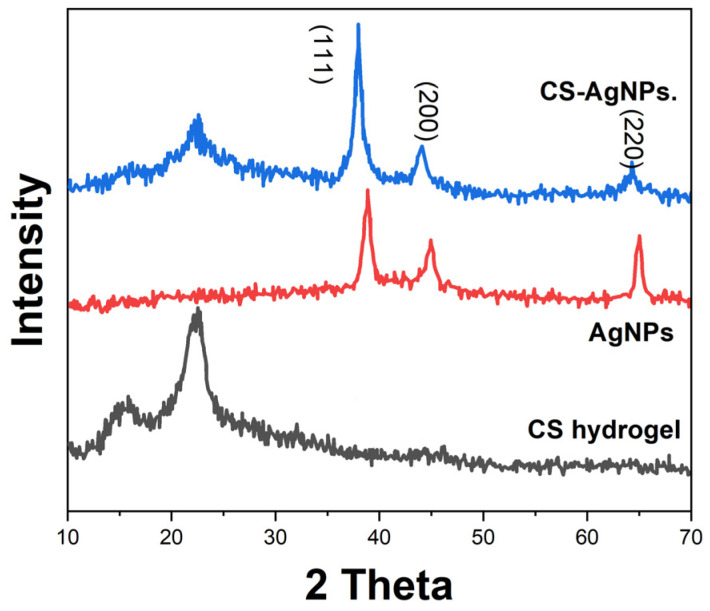
XRD patterns of CS hydrogel, AgNPs, and CS-AgNPs.

An X-ray diffraction (XRD, D8-Advance, Bruker, Billerica, MA, USA) analysis determined the crystalline phases of the components. Figure 3 shows the XRD patterns of cellulose, AgNPs, and the CS-AgNPs composite. The cellulose diffractogram contains a broad peak at 2θ = 22°, corresponding to the amorphous structure. In the AgNPs’ pattern, intense signals at 38°, 44°, and 64° and lattice planes verify the face-centered cubic geometry [31]. The composite pattern displays crystalline peaks of both cellulose and AgNPs, suggesting that nanocomposite formation leaves intact the individual components [32].

**Figure 4:**
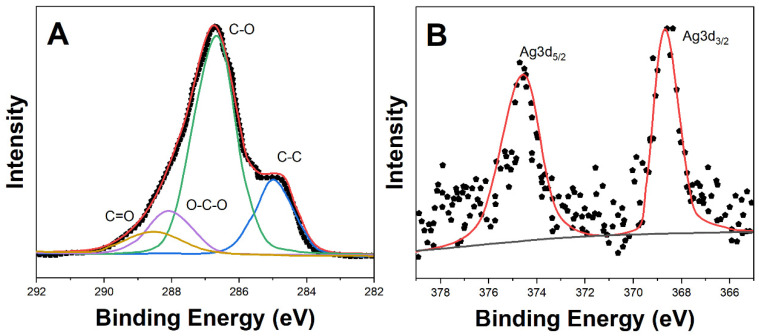
High-resolution XPS core-level spectra of (**A**) C1s peak of cellulose and (**B**) Ag3d peak of AgNPs present in the CS-AgNPs.

An XPS (K-Alpha, Thermo Fisher Scientific, Waltham, MA, USA) analysis provided insights into the surface chemical composition and oxidation states (Figure 4). High-resolution Ag3d core-level spectra show doublet peaks at 368.62 eV and 374.52 eV binding energies, attributed to Ag3d_5/2_ and Ag3d_3/2_ of silver atoms in the unoxidized state (Ag^0^). Kwon et al. [33] found similar results. The C1s spectrum was deconvoluted into four peaks at 284.9 eV, 286.7 eV, 288.0 eV, and 288.6 eV, assigned to the C-C, C-O, O-C-O, and C=O bonds of cellulose [34]. The instrument detection limit of <1 at.% silver verifies the uniform distribution of AgNPs within the cellulose matrix. A similar observation has been reported by Wang et al. [35].

**Figure 5:**
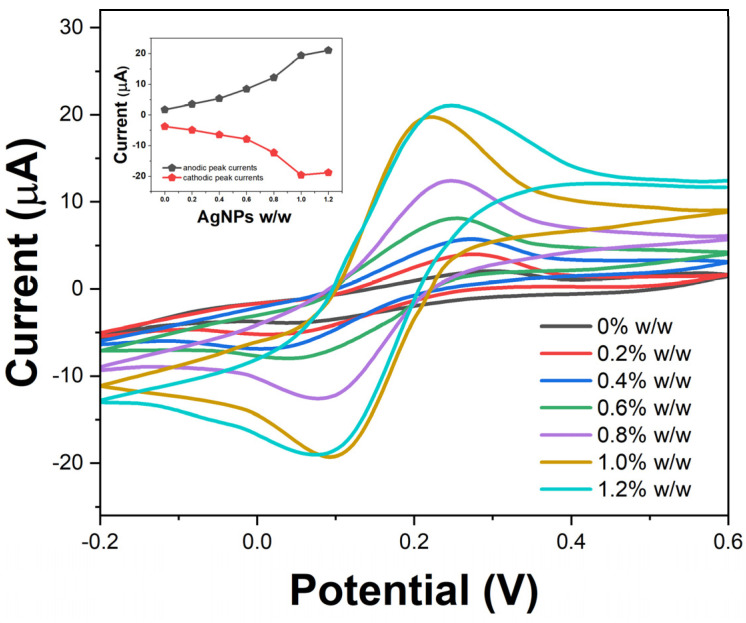
CV of disposable electrodes with different silver nanoparticle loadings in cellulose matrix. Inset shows effect on anodic/cathodic peak currents.

The electrochemical performance of the disposable sensors fabricated using the CS-AgNPs composite was evaluated by CV using potassium ferricyanide/ferrocyanide as a redox probe. Figure 5 shows the CVs obtained with varying concentrations of embedded AgNPs from 0 to 1.2% *w*/*w* at a scan rate of 50 mV/s. All voltammograms consist of a characteristic ferricyanide/ferrocyanide redox couple, indicating facile electron transfer kinetics at the sensor interface [36]. In the absence of any AgNPs, the cellulose-based electrode exhibits a peak-to-peak separation (ΔEp) of 220 mV, suggesting slow electron transfer on the bare cellulose matrix. As the concentration of AgNPs is increased, the ΔEp gradually decreases along with enhancement in the redox peak currents, indicating improved conductivity and charge transfer rates. This correlates with the inherent high electrical conductivity of AgNPs facilitating faster shuttling of electrons across the electrode interface [37]. At 1% *w*/*w* loading of AgNPs, the sensor demonstrates ΔEp of 125 mV, approaching a reversible electrochemical system.

**Figure 6:**
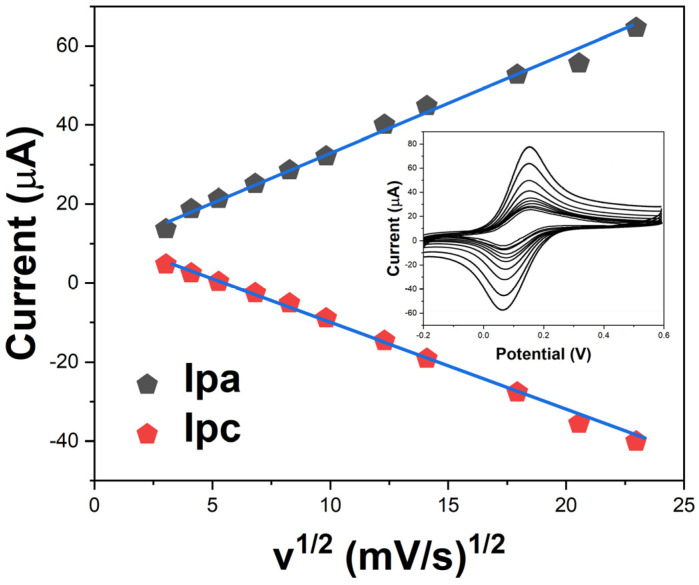
Plot of anodic/cathodic peak currents versus the square root of scan rate. Inset shows cyclic voltammograms at different scan rates.

The effect of the scan rate on the voltammetric response was investigated to understand the electrode reaction kinetics. Figure 6 shows that the redox peak currents vary linearly with the square root of the scan rate, affirming a diffusion-controlled electrode process. The electron transfer coefficient (α) was calculated to be 0.6, indicating fast heterogeneous charge transfer between the electrolyte and modified sensor interface [38]. These results verify the potential of the conductive cellulose–silver nanocomposite for enhancing performance of the disposable electrochemical sensors.

**Figure 7:**
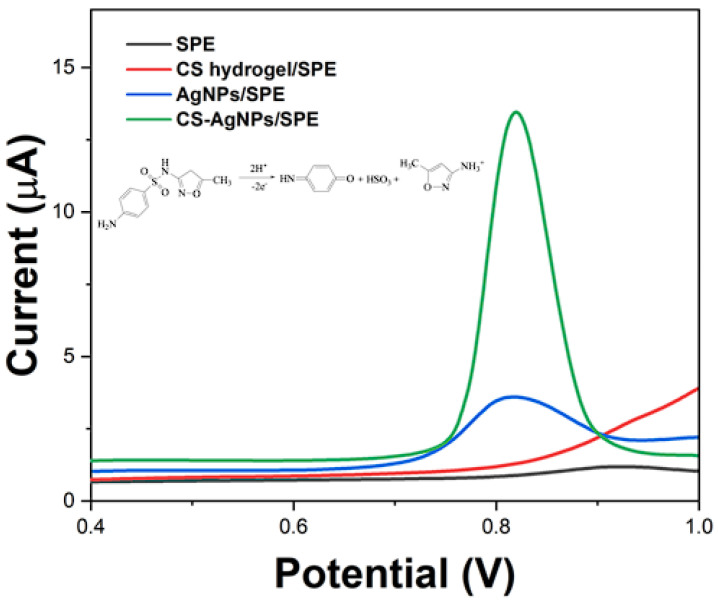
SWV of 10 μM SMX at SPE-, CS hydrogel-, AgNPs-, and CS-AgNPs-composite-modified sensor. Inset: structure and oxidation mechanism of SMX.

The SPE-, CS hydrogel-, AgNPs-, and CS-AgNPs-composite-modified sensors were explored for sensing SMX by SWV. Figure 7 displays the SWV profiles obtained with different sensors, showing a well-defined irreversible oxidation peak corresponding to the two-electron oxidation of SMX [39]. It can be seen that the CS-AgNPs-composite-modified sensor showed the highest peak current. A plausible mechanism involves initial formation of a radical cation intermediate followed by a second oxidation step leading to the iminoquinone product (inset of Figure 7) [40].

The voltammetric signals depend on instrumental parameters such as frequency, amplitude, and step potential, which can profoundly influence the sensitivity. By optimizing these parameters, the maximum peak current response was achieved at a frequency of 25 Hz, amplitude of 90 mV, and step potential of 4 mV. Furthermore, the effect of pH was screened between pH 2 and 8 using Britton–Robinson buffer medium. The highest current response was at pH 7, indicating favorable electrochemical oxidation in neutral conditions.

**Figure 8:**
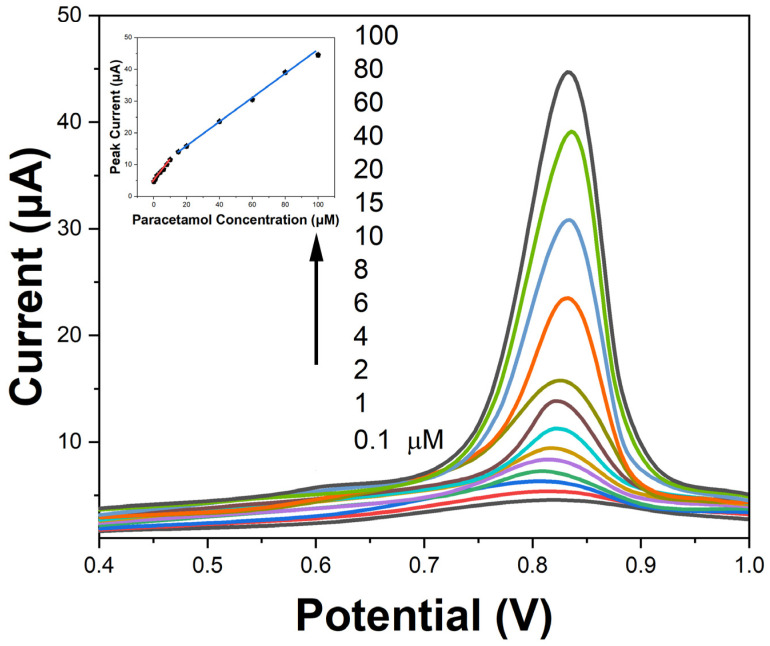
SWV obtained with increasing SMX concentrations. Inset: SWV peak current calibration plot for SMX at varying concentrations.

Under optimized SWV parameters, the sensor demonstrated two linear dynamic ranges, from 0.1–10 μM (R^2^ = 0.982) and 10–100 μM (R^2^ = 0.997) SMX concentrations, respectively, as shown by the calibration plot in Figure 8. The LOD was calculated to be 0.04 μM (10.13 μg SMX per kg) based on a signal-to-noise ratio S/N = 3. These analytical parameters are adequate to monitor SMX residues below the regulatory limits. The excellent dynamic range, high sensitivity, and low detection limits highlight the promising potential of the developed CS-AgNPs composite sensor for fast and facile screening of sulfonamide antibiotics in meat samples.

The applicability of the CS-AgNPs sensor was evaluated by spiked recovery experiments from meat samples. The minced meat was spiked with 10, 50, and 100 μM SMX standard solutions. SWV was performed after a simple extraction procedure, using perchloric acid followed by centrifugation and filtration of the supernatant. 

**Figure 9:**
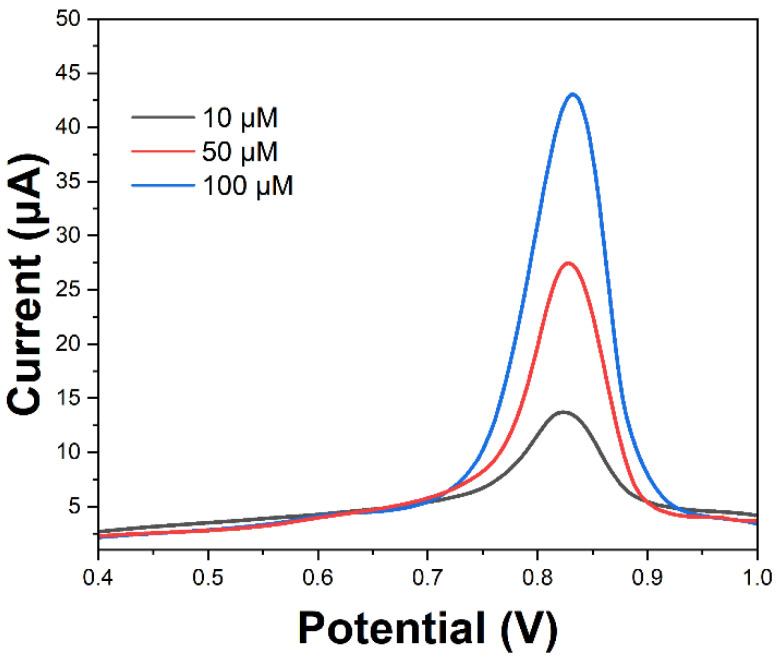
SWV profiles obtained after extracting spiked meat samples containing various SMX amounts.

Figure 9 shows the obtained voltammograms, indicating an increase in peak currents proportional to the SMX concentrations spiked in meat. This demonstrates the ability to successfully recover and detect the added SMX residues from the complex meat matrix. The peak currents showed negligible differences from the signals recorded using standard SMX solutions in buffer. This indicates the sensor selectivity and lack of fouling effects or interference from potentially oxidizable species present in real meat samples.

The extraction recovery percentages were determined by comparing the detected and added concentrations based on the calibration curve. Recoveries in the range of 86–92% were achieved, as shown in Table 1. The consistent recovery values validate the reliability of the proposed CS-AgNPs sensor for rapid voltammetric screening of sulfonamide drug residues in meat samples. Though we have demonstrated good recoveries from spiked minced meat, actual samples may pose additional matrix challenges. We posit the developed method will perform effectively for incurred residues since the extraction helps dissociate tissue-bound drugs while the centrifugation and filtration steps remove potentially interfering species from the meat matrix, as evident from the selectivity results. Additionally, the porous cellulose matrix facilitates molecular sieving on the sensor surface. These measures will likely enable reliable detection even with the residue levels and matrix interactions present in real samples.

**Figure 10:**
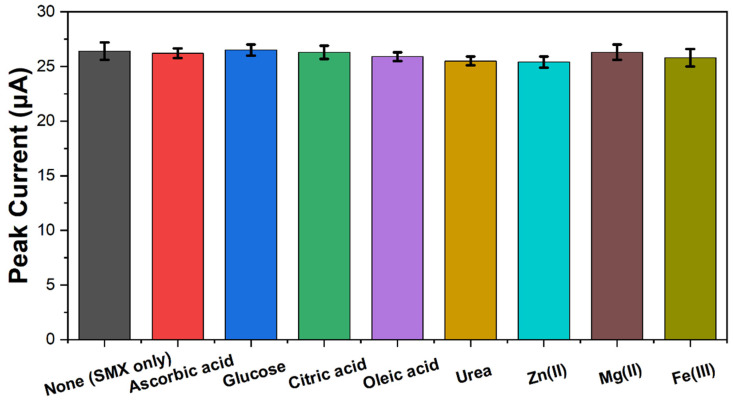
Bar chart showing comparative peak currents for 50 μM SMX in presence of 100 μM concentrations of various interfering species at pH 7 using the cellulose–silver nanoparticle sensor.

The reasonable reproducibility is indicated by an RSD below 6%. The produced CS-AgNPs/SPE was assessed by storing it under ambient conditions. Over a period of one month, no substantial decrease in the peak currents was observed, indicating excellent retention of conductivity and charge transfer kinetics. Furthermore, an interference study analyzed the SWV response of the 50 μM SMX standard with the addition of 100 μM levels of various possible interferences such as ascorbic acid, glucose, citric acid, oleic acid, urea, and ions like Zn^2+^, Mg^2+^, and Fe^3+^. As is evident in Figure 10, there were no significant shifts in the SMX oxidation peak position or current response even at twice the molar excess of interfering species. This demonstrates excellent anti-interference ability and selectivity of the developed sensor towards reliable quantification of SMX without pretreatment or separation steps. The porous cellulose matrix may facilitate molecular sieving and size-based exclusion of large protein and lipid molecules while the negatively charged hydroxyl groups can repel cationic species. Moreover, the strong coordination bonding between cellulose and AgNPs prevents leaching and provides a stable microenvironment at the sensor interface for unhindered electron transfer reactions involving SMX molecules.

The key analytical parameters obtained in this work using a CS-AgNPs composite sensor are compared to previous electrochemical sensors for SMX detection in Table 2. Most sensors rely on complex nanomaterials and time-consuming modification procedures to achieve low detection limits. In contrast, the present sensor offers comparative or superior detection limits and linear ranges without extensive fabrication steps or costs. This is ascribed to the high conductivity, surface area, and stability of the AgNPs embedded within the cellulose hydrogel matrix. Furthermore, the disposability, ease of mass production, lack of surface fouling, and matrix interferences make this sensor suitable for practical testing applications. Hence, the proposed sensor provides a promising cost-effective platform for rapid, on-site screening of SMX residues in meat samples. 

The main limitations of the proposed electrochemical sensor relative to established techniques include moderately lower sensitivity in the nanomolar range compared to the levels achievable using LC-MS or microbiological assays. Sample extraction and lack of chromatographic separation can pose selectivity challenges for complex real samples, leading to potential interferences. Additionally, the single-analyte detection capability is a disadvantage versus multi-residue analyses possible with advanced instrumentation.

## 3. Experimental

### 3.1. Chemicals and Reagents

All chemical reagents used in this work were of analytical grade and used without further purification. Graphite powder (product no. 282863; ≤20 μm), microcrystalline cellulose (product no. 310697; MCC, average particle size 50 μm), silver nitrate (product no. 209139), sodium borohydride (product no. 452882), potassium ferrocyanide (product no. P3289), polyvinylpyrrolidone (product no. PVP40), potassium ferricyanide (product no. 702587), and sulfamethoxazole (product no. 723-46-6) were procured from Sigma-Aldrich (St. Louis, MO, USA). Britton–Robinson buffer components (product no. 1631001), including boric acid, phosphoric acid, acetic acid, and sodium hydroxide pellets, were supplied by SDFCL (Mumbai, India). Methanol (product no. 10014108), acetone (product no. 10000418), and isopropyl alcohol (product no. 40064360) were obtained from Sinopharm Group Co., Ltd. (Shanghai, China). Deionized water (resistivity ≥ 18 MΩ cm) was used to prepare solutions. Polyethylene terephthalate sheets and all inks used for screen-printed electrode (SPE) construction were provided by Nanjing Youyun Biotech Co., Ltd. (Nanjing, China). The pork mince was procured from a local butcher shop without controlling for the fat content or composition. Five kilograms of pork mince was purchased for this study.

### 3.2. Synthesis of Silver Nanoparticles

The AgNPs were synthesized by the chemical reduction method [48]. A volume of 50 mL of aqueous solution containing 1 mM silver nitrate was heated to boiling under stirring. Then, 5 mL of 1% *w*/*v* sodium borohydride was added dropwise to reduce Ag+ ions. The solution was maintained at near boiling temperature for 1 h under vigorous stirring, resulting in a golden-brown colored colloid containing AgNPs. The particles were stabilized using a 5% *w*/*v* polyvinylpyrrolidone solution. The colloidal suspension was cooled to room temperature, centrifuged at 10,000 rpm (1341.7 g) for 15 min, and washed thrice with deionized water to remove excess reagents.

### 3.3. Preparation of Cellulose–Silver Nanoparticle (CS-AgNPs) Composite Hydrogel

A 1% *w*/*v* cellulose solution was prepared by dissolving MCC powder in 8% NaOH solution cooled to −6 °C using an ice bath. The AgNP colloid was added to achieve 0.5% loading (0.5 g AgNP colloid in 100 g sample) and homogenized (HX28K-48, Wenzhou Hongxiang Machinery Technology Co., Ltd., Wenzhou, China) by ultrasonication at 60% amplitude for 5 min. The mixture was cast as films in Teflon molds and kept overnight in a −20 °C deep freezer followed by thawing to induce gelation through hydrogen bonding of cellulose chains. The transparent cellulose–silver hydrogel films were washed in deionized water to remove residual alkali. Finally, the films were immersed in acetone for solvent exchange and dried at 60 °C for 12 h. The resultant aerogel films (CS-AgNPs) were used to fabricate disposable electrodes.

### 3.4. Development of Disposable Electrochemical Sensors

**Figure 11:**
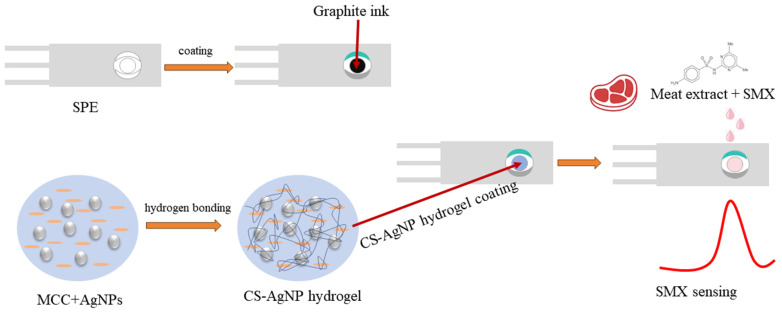
Schematic diagram of the preparation process of CS-AgNP-based disposable electrochemical sensor for SMX sensing.

A screen-printed three electrode system consisting of an Ag/AgCl reference, platinum counter, and carbon working electrodes on polyethylene terephthalate sheets was designed using appropriate masks. An 80:20 graphite–binder ink was screen-printed on the sheets and cured at 95 °C for 2 h. Next, a second layer of the cellulose–Ag NP composite hydrogel ink was printed on the graphite working area as the sensor interface and oven-dried. A dielectric ink was spotted to define the electrode areas and contact pads for measurements. The sheet, containing multiple electrode units, was cut into 4 mm width strips with an active working area of 12 mm^2^ to serve as single-use disposable sensors. Figure 11 shows a schematic diagram of the preparation process of such a disposable electrochemical sensor.

### 3.5. Electrochemical Measurements

All voltammetric measurements were performed using an Ivium Compactstat potentiostat. The disposable sensors were connected by an edge connector and the three electrode setup was completed by inserting a silver wire as a pseudo-reference electrode. For characterization studies, the electrodes were evaluated by recording cyclic voltammograms (CVs) in acetate buffer (pH 4) containing a 5 mM [Fe(CN)_6_]^3−/4−^ redox probe. Optimization of the experimental variables was performed to obtain well-defined and reproducible electrochemical signals. Various standard concentrations of SMX were prepared in 0.04 M Britton–Robinson buffer by serial dilutions. The square-wave voltammetry (SWV) technique was applied for analytical detection over the range of 0.1–100 μM by scanning from 0 to 1.2 V at 25 mV/s frequency. The measurements were carried out by drop-casting 70 μL samples onto the disposable sensor surface followed by incubation for 5 min before electrochemical scanning.

### 3.6. Preparation of Spiked Meat Samples

The meat samples were minced, homogenized, and stored at −20 °C until analysis. Two-gram portions of the homogenized meat were weighed into test tubes and spiked with SMX by adding standard solutions to obtain final concentrations of 10, 50, and 100 μg/kg. The samples were vortexed for 5 min and left to equilibrate for 1 h at room temperature [49]. Then, 7 mL of 0.1 M perchloric acid was added and homogenized for 5 min to dissociate the SMX residues. The extraction tubes were kept in an ice bath under ultrasonication at 40 kHz, 50% amplitude for 10 min. The extracts were centrifuged for 15 min at 10,000 rpm (1341.7 g). The supernatants were filtered using 0.2 μm PTFE syringe filters. Then, 1 mL aliquots were transferred to 10 mL tubes containing 1.5 mL of saturated sodium carbonate solution to neutralize excess acid. The neutralized extracts were reconstituted to 10 mL with 0.04 M B-R buffer forming the analysis samples, which were preserved at 4 °C until analysis by SWV as described earlier.

## 4. Conclusions

This work reports the development of a disposable electrochemical sensor using silver nanoparticle-embedded cellulose hydrogel composites for sensitive detection of sulfamethoxazole residues in meat samples. The cellulose matrix provided a porous biopolymer network to encapsulate conductive AgNPs of 20–50 nm size. Microstructural analyses by SEM and FTIR verified the uniform distribution of the AgNPs coordinated to cellulose chains through hydroxyl groups. The XRD and XPS results confirmed crystalline metallic AgNPs interacting with the amorphous cellulose hydrogel. Cyclic voltammetry using ferricyanide/ferrocyanide redox probe demonstrated enhanced conductivity and interfacial charge transfer kinetics arising from the embedded AgNPs facilitating faster electron shuttling. Square-wave voltammetry was utilized for sensing sulfamethoxazole based on its two-electron oxidation peak at 0.72 V vs. Ag/AgCl. After optimization, the sensor showed a dynamic range of 0.1–100 μM sulfamethoxazole, with a lower detection limit of 0.04 μM and sensitivity of 0.752 μA/μM in Britton–Robinson buffer of pH 7. The disposable sensor successfully recovered spiked sulfamethoxazole from meat samples in the range of 86–92% with less than 6% RSD, reflecting its practical reliability for real sample analysis. The key advantages of the proposed cellulose–silver nanocomposite sensor include facile fabrication, a lack of surface fouling, adequate sensitivity to meet regulatory requirements, and the potential for mass production of inexpensive disposable devices for decentralized on-site screening of antibiotic residues. Thereby, this research demonstrates a promising electroanalytical strategy using novel bionanocomposite materials towards rapid, low-cost monitoring to ensure food safety.

## Figures and Tables

**Table 1:** Recovery results from spiked meat samples.

Spiked SMX (μg/kg)	Detected (μg/kg)	Recovery (%)	RSD (*n* = 3)
10	8.6	86	5.2
50	46	92	3.8
100	92	92	1.9

**Table 2:** Comparison of analytical performance with other electrochemical SMX sensors.

Sensor Interface	Linear Range	Detection Limit (μM)	Reference
Graphitic carbon nitride and zinc oxide nanocomposite	20 nM–1.1 mM	6.6 nM	[41]
Ascorbic acid-reduced graphene oxide	0.5–50 μM	0.04 μM	[42]
Fe-doped ZnO nanorods	2.0–160.0 μM	30 nM	[43]
Carbon nanotube-modified antimony nanoparticles	0.1–0.7 μM	24 nM	[44]
Boron-doped diamond electrode	8.01–119 μM	1.15 μM	[45]
Graphene and ZnO nanorods	1–180 μM	0.4 μM	[46]
Ag/MWCNT	0.05–70 μM	0.01 μM	[47]
CS-AgNPs	0.1–100 μM	0.04 μM	This work

## Data Availability

The data that support the findings of this study are available on request from the corresponding author.
